# Supplementary material for: Paying Comprehensive Attention to the Temperature-Dependent Dual-Channel Excited-State Intramolecular Proton Transfer Mechanism of Fluorescence Ratio Probe BZ-DAM
Source: Int J Mol Sci. 2023 Sep 9;24(18):13899. doi: 10.3390/ijms241813899 (PMC10530469; doi:10.3390/ijms241813899)
Supplement: Supplementary file 1 [file ijms-24-13899-s001.zip › ijms-2545684-supplementary.pdf]

Paying Comprehensive Attention to the Temperature-Dependent Dual-Channel Excited-State Intramolecular Proton Transfer Mechanism of Fluorescence Ratio Probe BZ-DAM

Jiaan Gao, Yifu Zhang, Hongyan Mu, Min Yang, Xiaotong Guan, Guangyong Jin * and Hui Li *

Jilin Key Laboratory of Solid-State Laser Technology and Application, School of Physics,Changchun University of Science and Technology, Changchun 130022, China

** To whom correspondence should be addressed. E-mails: huili@cust.edu.cn and jgycust@163.com*

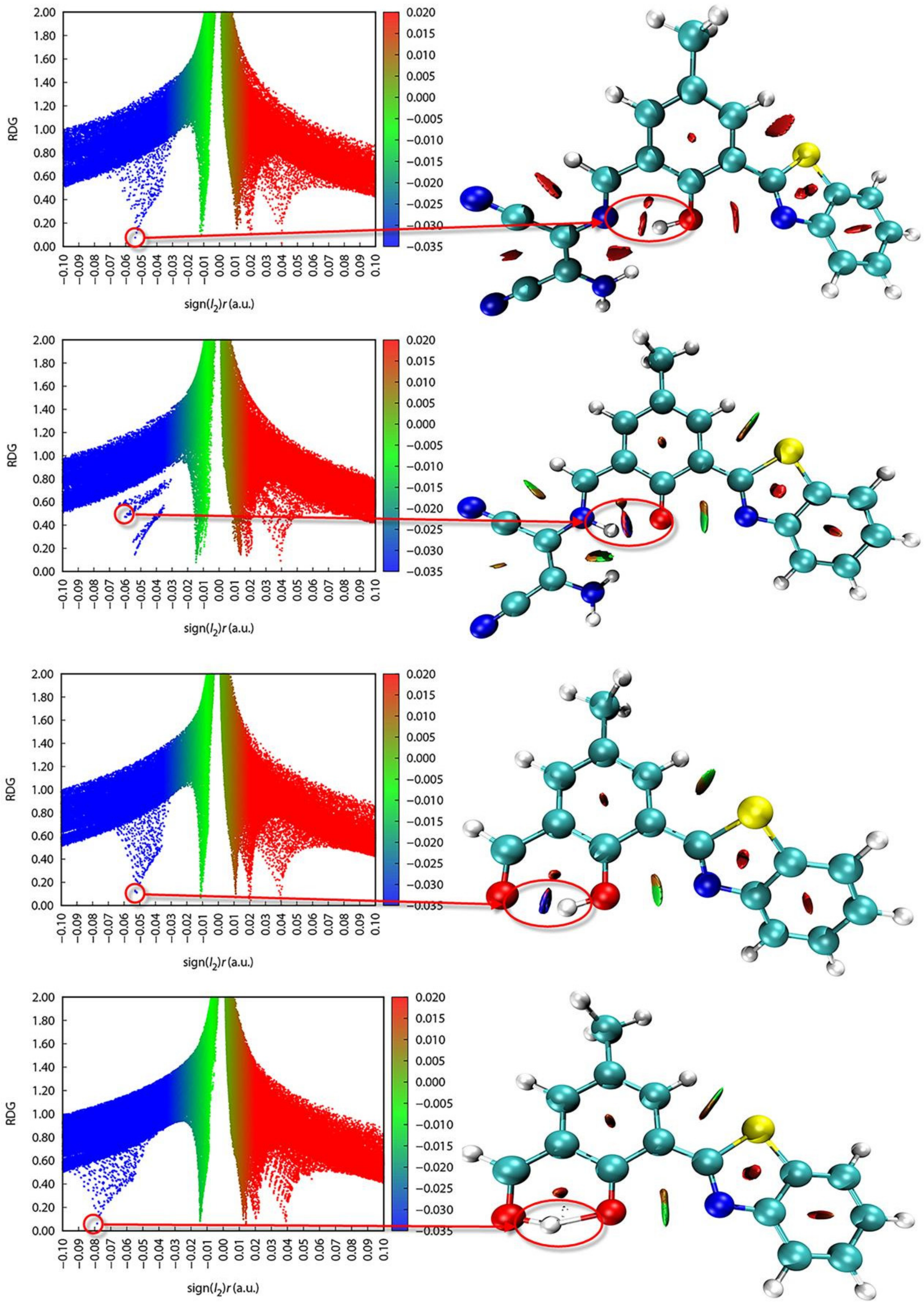

Figure S1. RDG versus Sign(λ_2) ϱ(r) scatter plots of BZ-DAM and BZ-CHO in channel 2 in the S_0 and S_1 states.

Table S1 The structures and properties of three kinds of fluorescent probes reported are compared.

type	Probes	Detection mechanism	Ref.
"turn-off" fluorescent probe	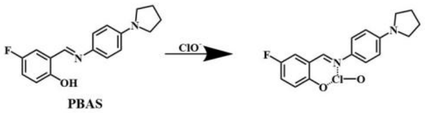	Reduction in fluorescence intensity	J Fluoresc. **2019**, 29, 399-406
	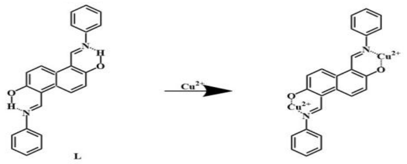	Reduction in fluorescence intensity	Molecules. **2021**, 26, 1-11
	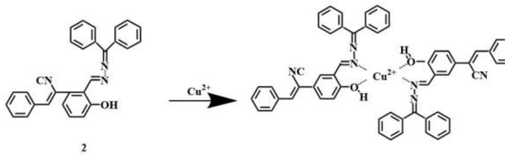	fluorescence quenching	Inorg. Chem. Commun. **2023**, 152, 110640
	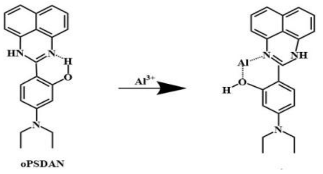	fluorescence quenching	Spectrochim. Acta. Part A. **2023**, 293, 122471
	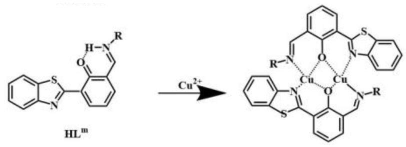	fluorescence quenching	Spectrochim. Acta. Part A. **2023**, 287, 122051
"turn-on" fluorescent probe	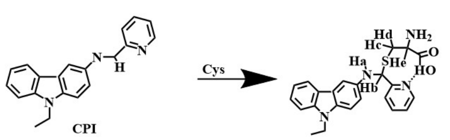	increase in fluorescence intensity	Analytical Bioanalytical Chem. **2019**, 411, 6203–6212
	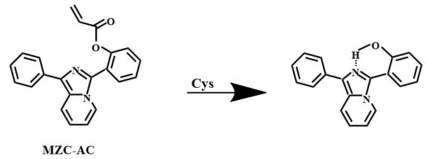	increase in fluorescence intensity	ACS Omega. **2020**, 5, 19695–19701
	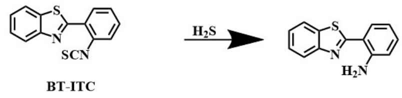	increase in fluorescence intensity	Spectrochim. Acta. Part A. **2022**, 278, 121333z
	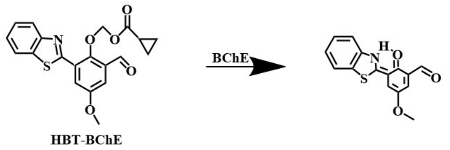	increase in fluorescence intensity	Spectrochim. Acta. Part A. **2023**, 287, 122044
	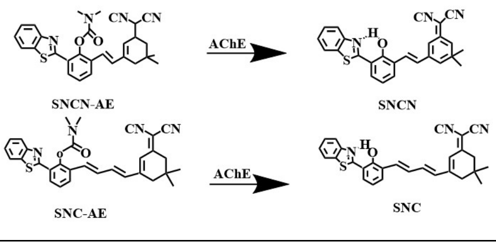	increase in fluorescence intensity	Sens. Actuators B Chem. **2023**, 380, 133392

type	Probes	Detection mechanism	Ref.
	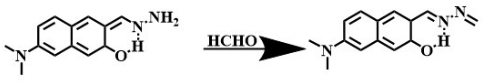 Naph-1	increase in fluorescence intensity	Analyst. **2019**, 144, 6922
	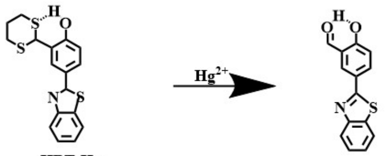 HBT-Hg	Fluorescence red-shift	Spectrochim. Acta. Part A. **2020**, 243, 118817
Ratio fluorescent probe	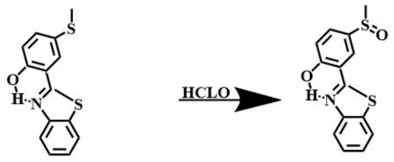 BTMSP BTMTP	Fluorescence blue-shift	Theor Chem Acc. **2022**, 141, 57
	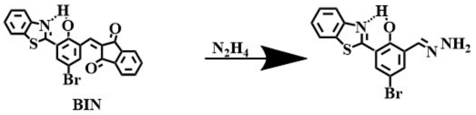 BIN	Fluorescence blue-shift	Anal. Chim. Acta. **2022,** 1227, 340320
	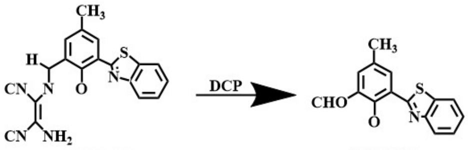 BZ-DAM BZ-CHO	Fluorescence blue-shift	New J. Chem. **2023**, 47, 250

Table S2 Calculated transition properties of BZ-DAM and BZ-CHO in ACN solvent at the TD-DFT/mpw1pw91/6-31g (d, p) theoretical level.

		transition	λabs(nm)	f	Composition	CI (%)
channel 1	BZ-DAM	$S_0 \to S_1$	397.15	0.6100	H→L	64.20%
		$S_0 \to S_2$	342.38	0.3723	H-1→L	65.03%
		$S_0 \to S_3$	337.50	0.0677	H→L+1	68.06%
		$S_0 \to S_4$	309.68	0.4493	H-1→L+1	66.94%
		$S_0 \to S_5$	298.45	0.0003	H-4→L	67.56%
		$S_0 \to S_6$	297.15	0.0772	H-2→L	64.82%
	BZ-CHO	$S_0 \to S_1$	345.78	0.4650	H→L	69.33%
		$S_0 \to S_2$	333.43	0.0000	H-3→L	51.68%
		$S_0 \to S_3$	292.50	0.0396	H-1→L	58.32%
		$S_0 \to S_4$	285.48	0.2130	H→L+1	45.52%
		$S_0 \to S_5$	276.51	0.0839	H-2→L+1	51.72%
		$S_0 \to S_6$	268.22	0.0001	H-3→L+1	49.82%
channel 2	BZ-DAM-1	$S_0 \to S_1$	399.81	0.6100	H→L	66.28%
		$S_0 \to S_2$	350.61	0.5187	H-1→L	66.71%
		$S_0 \to S_3$	323.33	0.0012	H-2→L	69.92%
		$S_0 \to S_4$	309.98	0.1207	H→L+1	65.72%
		$S_0 \to S_5$	298.23	0.0353	H-3→L	63.81%
		$S_0 \to S_6$	287.25	0.3763	H-1→L+1	65.42%
	BZ-CHO-1	$S_0 \to S_1$	346.36	0.2692	H→L	69.37%
		$S_0 \to S_2$	305.02	0.0006	H-3→L	55.00%
		$S_0 \to S_3$	299.94	0.0094	H-1→L	66.56%
		$S_0 \to S_4$	285.00	0.2161	H→L+1	56.15%
		$S_0 \to S_5$	282.60	0.1481	H-2→L	55.75%
		$S_0 \to S_6$	265.35	0.0312	H-1→L+1	63.46%

Table S3 Indexes characterizing the distribution of choles and celes of BZ-DAM and BZ-CHO.

	D(Å)	Sm	Sr	H(Å)	t(Å)
BZ-DAM-S_1	0.658	0.476	0.751	3.545	-1.987
BZ-DAM-S_1'	1.507	0.387	0.673	3.744	-1.451
BZ-CHO-S_1	0.242	0.407	0.675	3.159	-1.329
BZ-CHO-S_1'	1.135	0.381	0.684	2.909	-1.148
BZ-DAM-1-S_1	3.960	0.276	0.595	3.399	1.441
BZ-CHO-1-S_1	2.525	0.364	0.678	2.958	0.150
